# Endogenous endophthalmitis caused by *Streptococcus suis* infection: a case report

**DOI:** 10.1186/s12886-022-02389-9

**Published:** 2022-04-09

**Authors:** Zhe Li, Min Xu, Xin Hua

**Affiliations:** Department of Ophthalmology, Subei Peoples’ Hospital Affiliated to Yangzhou University, Yangzhou, China

**Keywords:** Endophthalmitis, Meningitis, *Streptococcus suis*, Next generation sequencing

## Abstract

**Background:**

*Streptococcus suis* (*S. suis*) is an emerging zoonotic human pathogen, which commonly causes meningitis and sepsis. Ocular infections associated with *S. suis* infection are very rare. Herein, we reported a rare case of a man who developed endophthalmitis complicated by meningitis following *S. suis* infection.

**Case presentation:**

A 48-year-old male with a fever, loss of vision in the right eye, slight headache, and hearing loss in the right ear was admitted to our hospital. A comprehensive ophthalmological examination suggested endophthalmitis. The metagenomics next-generation sequencing (mNGS) results of pathogenic microorganisms from vitreous and cerebrospinal fluid samples revealed that the causative pathogen was *S. suis*, which was further confirmed by the bacterial culture of the vitreous sample. Subsequently, the patient received phacoemulsification and vitrectomy, combined with silicone oil tamponade, as well as local and systemic anti-infective therapy, after which his condition significantly improved.

**Conclusions:**

Despite the low incidence rate of endophthalmitis caused by *S. suis*, clinicians should be aware of relevant clinical manifestations, especially for patients with neurological symptoms and risk factors for *S. suis* infection. The next-generation sequencing is efficient for etiological diagnosis of pathogenic microorganisms.

## Background

*Streptococcus suis* (*S. suis*) is an emerging zoonotic human pathogen. Since the first reported case of human *S. suis* infection in Denmark in 1968, more than 1600 sporadic cases have been reported worldwide [1]. In China, two severe outbreaks were reported in Jiangsu and Sichuan Province in 1998 and 2005, respectively [2]. The bacteria are usually found in the upper respiratory tract (mainly the tonsil and nasal cavity), gastrointestinal tract, and genitalia of pigs [1]. Direct contact with infected animals, which usually occurs through the skin or mucosal wounds, may cause meningitis, septicemia, endocarditis, and other diseases in humans, such as enteritis, arthritis, endocarditis, pneumonia, spondylitis, endophthalmitis, and peritonitis [3]. Ocular infections associated with *S. suis* infection are very rare.

Herein, we reported on a rare case of endogenous endophthalmitis accompanied by atypical symptoms of central nervous system infection caused by *S. suis* that was detected by metagenomic next-generation sequencing (mNGS).

## Case presentation

**Fig. 1:**
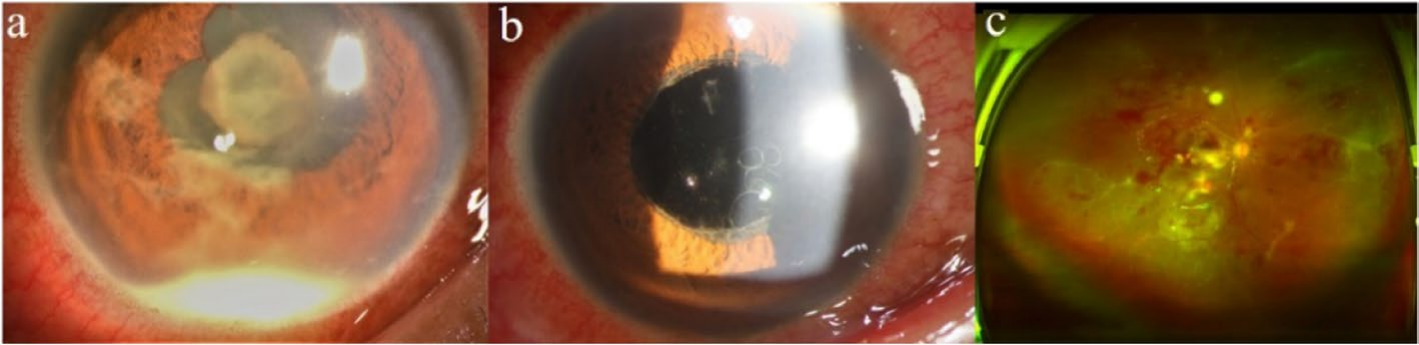
Anterior segment and fundus before and after the operation. **a** The anterior segment before operation showing a 2 mm white hypopyon and fibrinous exudative emma in the pupil covering the lens with no visible fundus. **b** and **c** Eight days after surgery, no hypopyon was seen in the anterior chamber, and the fundus was visible

A 48-year-old male who suffered from fever for 3 days accompanied by vision loss in the right eye for 2 days, was admitted to a local hospital. After intravenous antibiotic therapy, the patient’s symptoms worsened and the patient was referred to our hospital. He reported having fever and headaches for 2 days before the referral, with hearing loss in the right ear and pain, redness, and decreased vision in the right eye. His medical history revealed hypertension and no history of trauma or surgery. He was conscious, with normal body temperature, and neurological examinations were normal. The best-corrected visual acuity (BCVA) of the right eye was reduced to light perception, and the intraocular pressure was 21 mmHg. A 2-mm white hypopyon and fibrinous exudative membrane in the pupil were observed. Vitreous and fundus could not be visualized (Fig. 1a). The left eye was unaffected. A heterogenous vitreous echogenicity appeared on B-scan ultrasonography. No abnormalities were found on the orbital computed tomography (CT) scan, cranial magnetic resonance imaging (MRI), and upper abdomen CT.

White blood cell count, neutrophils, monocytes, and platelets were all in the normal range, while the erythrocyte sedimentation rate (ESR) was 37 mm/hr. and C-reactive protein was 40.68 mg/L. After admission, a lumbar puncture was performed, and the cerebrospinal fluid (CSF) pressure was 280 mm H_2_O (normal: 80 ~ 180 mm H_2_O). A routine CSF test showed a white blood cell of 55 × 10^6^/ L and monocytes of 53 × 10^6^/ L. The positive Pandy’s test was in accordance with the CSF biochemical results, revealing an elevated protein of 1.48 g/L. The electro-audiometer detected a hearing loss in both ears, and moderate loss in the right ear. Thus, the initial diagnosis was endogenous endophthalmitis in the right eye and meningitis.

Immediately after admission, we performed an anterior chamber tap. The patient was also given an intravitreal injection of antibiotics (0.1 ml of 10 g/L vancomycin, 0.1 ml of 20 g/L ceftazidime) in the right eye. The obtained aqueous humor and blood sample were sent for culture. On the second day, phacoemulsification and 23G pars plana vitrectomy (PPV) combined with silicone oil tamponade were performed. In addition, the vitreous biopsy was obtained for culture before PPV. Also, mNGS of pathogenic microorganisms were performed for vitreous and CSF biopsy, which is the most widely used for pathogen detection in infectious disease, could detect all pathogens nucleic acids of the samples, and the main procedures include nucleic acid extraction, library generation, sequencing and bioinformatics analyses [4]. Within 24 h of admission, the patient was prescribed 2 g of intravenous ceftriaxone twice per day and local corticosteroid, antibiotic, and mydriatics therapy. Three days later, *S. suis* was detected in both vitreous and CSF biopsy by mNGS. Five days after the surgery, the positive culture result of the vitreous biopsy was in accordance with the mNGS, and the antibiotic susceptibility test results showed it was susceptibility to chloramphenicol (MIC≤4 μg/mL), cefotaxime (MIC≤1 μg/mL), vancomycin (MIC≤1 μg/mL), levofloxacin (MIC≤2 μg/mL), penicillin (MIC≤0.12 μg/mL), and resistance to erythromycin (MIC≥1 μg/mL), tetracycline (MIC ≥8 μg/ mL). Yet, the blood culture was negative. Finally, the patient was diagnosed with endogenous endophthalmitis and meningitis caused by *S. suis* infection.

After PPV and two-weeks effective anti-infective treatment, the infection was brought under control, no hypopyon was seen in the anterior chamber, and the fundus was visualized (Fig. 1b and c). Headache as well as the hearing loss in the left ear improved. Visual acuity in the right eye was HM/BE. Two weeks later, the CSF pressure was relieved to 215 mmH_2_O. Routine CSF test showed the white blood cell of 21 × 10^6^/ L and monocytes of 21 × 10^6^/ L. Decreased protein was detected in CSF from 1.48 g/L to 0.75 g/L. At 8-month follow-up, the clinical examination showed no inflammation of anterior chamber or vitreous of the right eye, the visual acuity maintained HM/BE and intraocular pressure was normal.

## Discussion and conclusion

Endophthalmitis caused by *S. suis* has been rarely reported. Meningitis associated with *S. suis* infection accounts for 84.6 and 75.2% among European and Asian populations, respectively, while sepsis accounts for 15.4 and 18.6%, respectively [3]. McLendon reported the first case of endophthalmitis caused by *S. suis* in 1978 [5]. The main manifestations were binocular endophthalmitis combined with right sensorineural deafness and impaired vestibular function. In 2019, Ajaree Rayanakorn reported another case in a middle-aged Thai man infected with *S. suis* after eating raw pork, which resulted in endogenous endophthalmitis of the right eye, accompanied by sepsis, infectious spondylitis, and meningitis [6]. The patient’s right eye was enucleated because of a severe ocular infection. His initial symptoms were chills, fever, mild headache, vision loss in the right eye, and hearing loss in the right ear.

Our patient visited the ophthalmology department for severe ocular symptoms and was initially diagnosed with endophthalmitis in the right eye. Although accompanied by chills, fever, and mild headache at that time, no obvious signs of central nervous system infection were observed after physical examination or cranial MRI. Consequently, the *S. suis* was confirmed by the mNGS technique.

People working with pigs or pork, such as pig farmers, slaughterhouse workers, meat processors, and veterinarians, are considered a high-risk group for *S. suis* infection [7]. Our patient works as a construction contractor in Shanghai. However, he did not travel to anywhere and had no pig farming exposure prior to admission at the hospital. The transmission route of infection in our patient remains unclear.

The negative blood culture results may be related to the sensitivity of *S. suis* to most antibiotics which were administrated prior to the culturing. For this patient, the pathogen was identified using mNGS in vitreous fluid and CSF, which was faster than positive bacterial culture result of vitreous fluid. Detection of pathogenic bacteria by this method is not limited due to the use of broad-spectrum antibiotics, as well as the presence of bacteria that are fastidious or slow growing [8]. However, the rate of false-positive by mNGS was higher than that by conventional methods. Thus, it is important for clinicians to distinguish between true- and false-positive pathogenic bacteria [9]. Although many technical challenges remain to overcome for its use, it still will be a useful mean for clinicians to achieve better clinical diagnosis and treatment.

Despite the low incidence of endophthalmitis caused by *S. suis*, clinicians should be aware of relevant clinical manifestations, especially in patients with neurological symptoms and high-risk factors for *S. suis* infection.

## Data Availability

All the data supporting the conclusions of this article are included in the present article.

## Abbreviations

mNGS: Metagenomics next-generation sequencing
BCVA: Best-corrected visual acuity
HM/BE: Hand motion before eye
ESR: Erythrocyte sedimentation rate
CSF: Cerebrospinal fluid
PPV: Pars plana vitrectomy
